# Diagnostic test accuracy of new generation tympanic thermometry in children under different cutoffs: a systematic review and meta-analysis

**DOI:** 10.1186/s12887-020-02097-7

**Published:** 2020-05-12

**Authors:** Dan Shi, Li-Yuan Zhang, Hai-Xia Li

**Affiliations:** Nursing Department, Hospital Affiliated 5 to Nantong University (Taizhou People’s Hospital), 366 Taihu Road, Medical High-tech district, Taizhou, Jiangsu Province China

**Keywords:** Tympanic thermometry, Pediatric, Rectal, Cutoff, Sensitivity, Specificity

## Abstract

**Background:**

The infrared tympanic thermometer (IRTT) is a popular method for temperature screening in children, but it has been debated for the low accuracy and reproducibility compared with other measurements. This study was aimed to identify and quantify studies reporting the diagnostic accuracy of the new generation IRTT in children and to compare the sensitivity and specificity of IRTT under different cutoffs and give the optimal cutoff.

**Methods:**

Articles were derived from a systematic search in PubMed, Web of Science Core Collection, and Embase, and were assessed for internal validity by the Quality Assessment of Diagnostic Accuracy Studies-2 (QUADAS-2). The figure of risk of bias was created by Review Manager 5.3 and data were synthesized by MetaDisc 1.4.

**Results:**

Twelve diagnostic studies, involving 4639 pediatric patients, were included. The cut-offs varied from 37.0 °C to 38.0 °C among these studies. The cut-off 37.8 °C was with the highest sROC AUC (0.97) and Youden Index (0.83) and was deemed to be the optimal cutoff.

**Conclusion:**

The optimal cutoff for infrared tympanic thermometers is 37.8 °C. New Generation Tympanic Thermometry is with high diagnostic accuracy in pediatric patients and can be an alternative for fever screening in children.

## Background

Body temperature measurement is a routine in the management of sick children for both parents and healthcare providers [1, 2]. An accurate diagnosis of fever is crucial in clinical practice [3, 4] and an inaccurate one could lead to serious complications and improper medical decisions [3, 5]. Core temperature is the gold standard for temperature measurement [3]. However, core temperature measurements, such as pulmonary artery and lower esophagus measurement, are invasive and require specialized equipment, therefore, are unpractical for daily clinical practice [3, 6]. Ideally, body temperature measurement should be noninvasive, accurate, pain-free, cost-effective and time-efficient [3, 7, 8].

Traditionally, non-invasive methods of body temperature measurement include rectal temperature, oral temperature and axillary temperature. Among these methods, rectal thermometry has been the most reliable for measuring body temperature in children and is considered clinically to be the best estimation of the core temperature [9]. However, it is time-consuming and requires certain level of practice [5, 10]. Furthermore, it may cause emotional distress, and -although very rare- brings possible complications such as perforation or transmission of micro-organisms [5, 10]. And therefore infants, health workers and parents more or less express reluctance to perform it [3].

The forehead skin thermometer (FST) and infrared tympanic thermometer (IRTT) are popular alternatives for the traditional measures. The FST uses a sensor probe to measure the amount of infrared heat produced by the temporal arteries [8]. The IRTT detects the radiation of tympanic membrane and the ear canal, which share the blood supply with the hypothalamus, the thermoregulatory center of the human body [11, 12]. Both these two methods are safe, easy to use, comfortable and quick. But compared to the FST, the IRTT is more consistent with rectal temperature and is more convincing [3, 8, 13]. Using the aural temperature is less traumatic and allows a faster triage [14], but it has been debated for the low accuracy and reproducibility compared with other measurements [1, 14–18]. Over the past years, however, the IRTT have been developed and updated, and some older versions have been obsolete. The new generation IRTT used various brand-specific ways to enhance accuracy, for example, improvements of geometry and algorithms, a wider angle measurement, displaying temperature on multiple samples and equipping with a heat probe [11, 19]. Synthesizing studies applying obsolete IRTT with the new ones is unreasonable and may underestimate the IRTT test accuracy. Furthermore, the cutoffs of the IRTT used in fever detection are diverse, and the optimal cut-off has no consensus. The cutoff means a temperature threshold that divides pediatric patients into fever and non-fever, and the diagnostic accuracy of IRTT various under different cutoffs [3, 13, 20, 21]. It is inappropriate to synthesize studies applying different cutoffs and the results are unreliable.

The aims of this systematic review were (1) to identify and quantify studies reporting the diagnostic accuracy of the new generation of the IRTT in children (By new generation, we meant the IRTT that were still in production and on sale according to the official websites of the manufacturers as we started our study); (2) to compare the sensitivity and specificity under different cutoffs of the IRTT and give the optimal cutoff.

## Methods

### Search strategies

The conduct of this systematic review and meta-analysis was based on the Test Accuracy Working Group of the Cochrane Collaboration and the Preferred Reporting Items for Systematic Reviews and Meta-Analyses of Diagnostic Test Accuracy Studies statement (The PRISMA-DTA Statement) guidelines [22, 23]. A systematic literature search of multiple electronic databases (PubMed, Web of Science Core Collection, EMBASE) was conducted by two trained reviewers (D.S. and LY.Z.) independently from inception to February 2nd, 2019. The following search terms ((tympanic thermometer OR ear thermometer OR infrared thermometry OR ear thermometry OR tympanic scan OR tympanic temperature OR ear temperature OR infrared thermometer OR ear thermometer)) AND (pediatric OR child OR kid OR newborn OR baby OR infant OR toddler) in All Fields (PubMed, EMBASE) or Topic (Web of Science Core Collection) were used. The languages were restricted to English and species were restricted to humans. The bibliographies of included studies were also searched to identify additional studies.

### Study selection

Observational studies, detecting fever by aural and rectal thermometers, were deemed acceptable. Inclusion criterion included (1) studies recruiting pediatric subjects (age < 18 years), (2) diagnostic test accuracy studies, (3) studies detecting fever by new generation IRTT, and (4) studies using rectal thermometers as the reference standard. Exclusion criterion included (1) studies unrelated to the accuracy of IRTT, (2) reviews, proceedings papers, meeting abstracts, letters, notes and editorial materials, and (3) studies lacking essential data.

Two reviewers (D.S. and LY.Z.) independently reviewed the titles and abstracts of these studies. Papers deemed to match the predefined inclusion criteria or without consensus were reviewed in full text. Disagreements were resolved through discussions and scientific consultations.

### Quality assessment and data extraction

We adopted the Quality Assessment of Diagnostic Accuracy Studies-2 (QUADAS-2, [24] for quality assessment and used Review Manager 5.3 for creating the figures of risk of bias and applicability concerns [25]. Two independent reviewers (D.S. and LY.Z.) assessed the methodological quality of the included studies independently and disagreements were also resolved through discussions and scientific consultations.

The following data were extracted by two independent reviewers (D.S. and LY.Z) from the included studies: (1) descriptive aspects: primary author, year of publication, country, setting, age, types of tympanic thermometer and reference standard; (2) statistical aspects: the size, number of observations, the cut-off of tympanic thermometer, the True Positive (TP), the False Negative (FN), the False Positive (FP) and the True Negative (TN), sensitivity, specificity, positive predictive value (PPV) and negative predictive value (NPV).

### Statistical analysis

Meta-analyses of TP, FN, FP and TN were performed to compare the test accuracy between tympanic temperature and the gold standard (rectal temperature) by MetaDiSc 1.4 [26]. Threshold analysis was conducted to evaluate the threshold effect [27]. The inconsistency index (I^2^) test was used to estimate heterogeneity between studies and I^2^ > 75% was considered to be with high heterogeneity [28]. Data were synthesized by using the random-effects model which was recommended in pooled estimates of diagnostic meta-analyses [29]. The area under the curve (AUC), Youden index and index Q* were used to measure test accuracy [30–32].

## Results

### Selection process

**Fig. 1:**
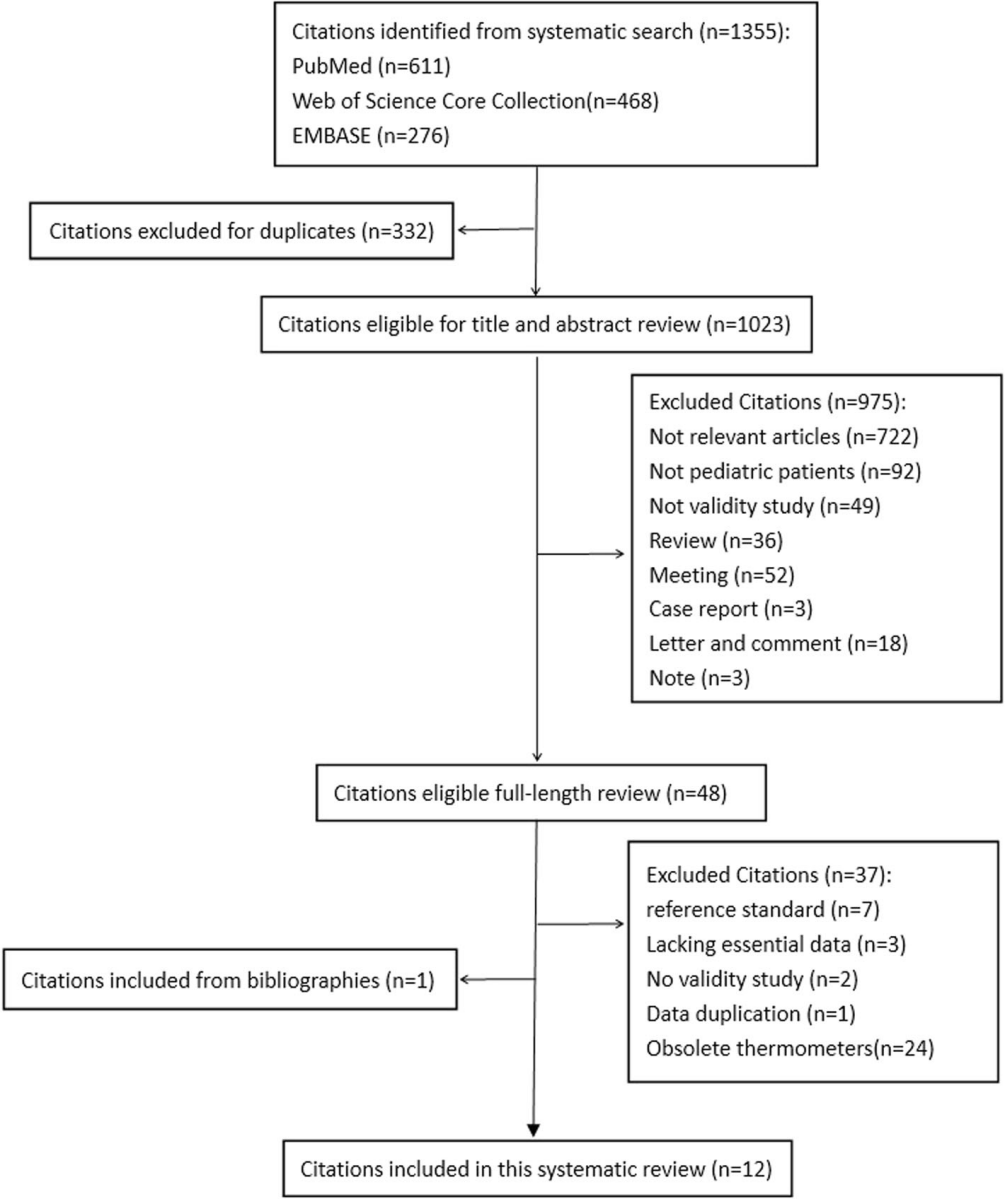
Study flow diagram of study selection process

Initially, 611, 468 and 276 articles were retrieved from PubMed, Web of Science Core Collection and EMBASE respectively. Secondly, 332 duplicates were removed. Thirdly, the titles and abstracts of the remaining 1023 articles were examined and 975 articles were excluded for diverse reasons. Finally, 11 articles were selected after the full text review and 1 article [33] was added by reviewing references. The process and outcome of the literature selection are presented in detail in Fig. 1.

### Risk of bias and applicability concerns in included studies

**Fig. 2:**
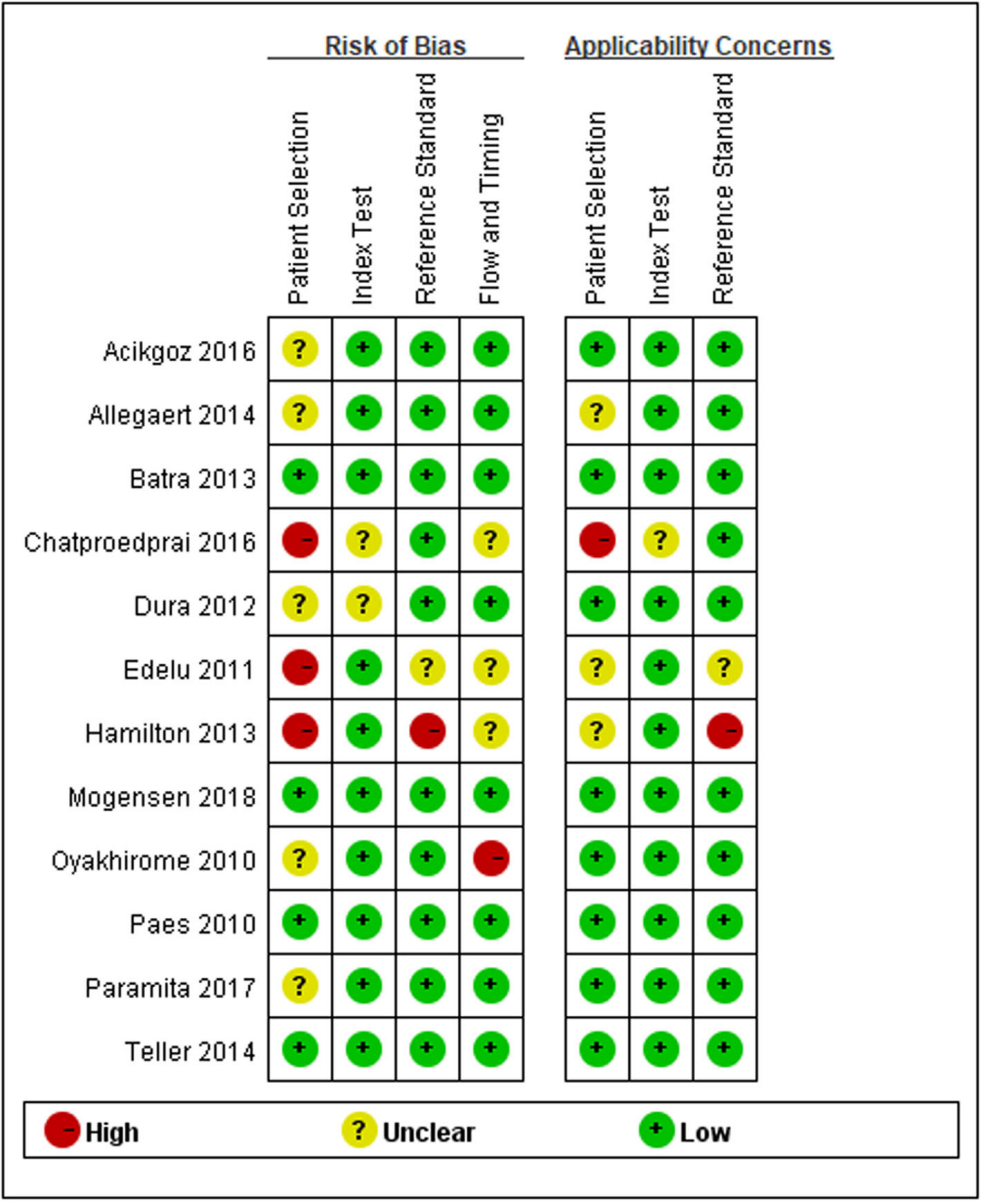
Outcomes of quality assessment of each included studies (by QUADAS-2)

**Fig. 3:**
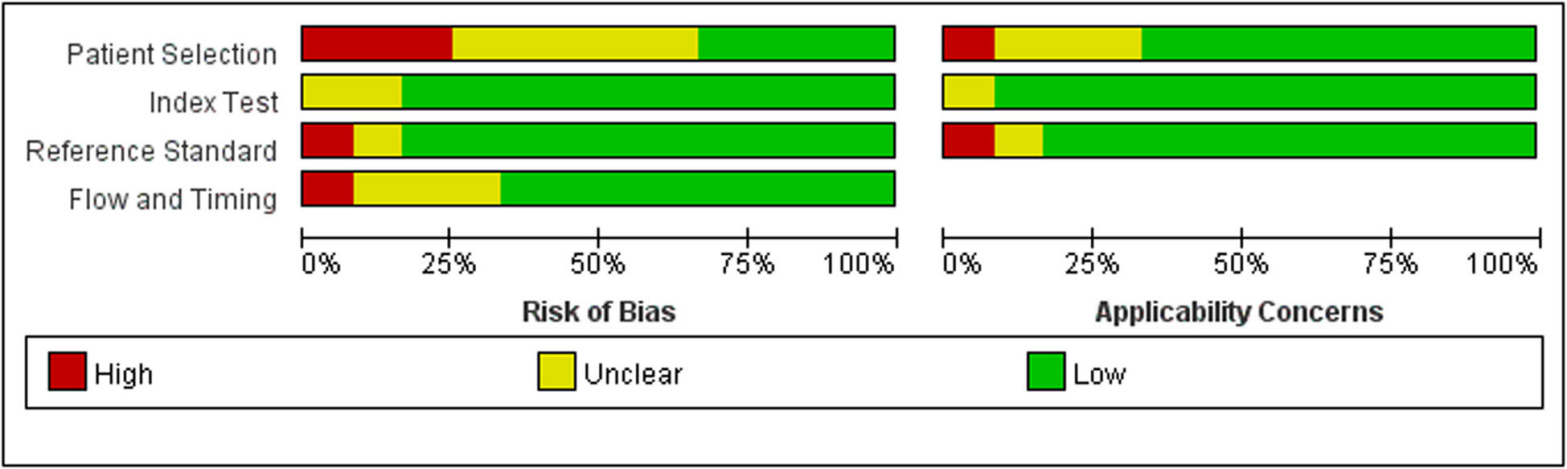
Overall quality assessment of included studies (by QUADAS-2): proportion of studies with low, unclear, and high risk of bias (left), and proportion of studies with low, unclear, and high concerns regarding applicability (right)

Figure 2 and Fig. 3 showed the risk of bias and applicability concerns in different domains. Among these 12 included articles, 4 had a high risk of bias on “flow and timing”, “patient selection”, “index test”, and “reference standard”, indicting the quality Methodological quality of included studies was moderate. Eight out of twelve studies had low applicability concerns in all domains and the applicability concerns was low.

### Characteristics of selected studies

Twelve included studies were published from 2010 to 2018. All these studies applied the tympanic thermometer and set the rectal thermometer as reference standard. The descriptive and statistical characteristics of the 12 studies were presented in Table 1 and Table 2 respectively.

**Table 1:** Descriptive characteristics of including studies

Studies	Year	Country	Setting	Age	Tympanic thermometer	Reference standard
Mogensen et al [13]	2018	Denmark	pediatric emergency department	0-18y	Braun Welch Allyn Pro 4000 Thermoscan	Rectal
Paramita et al [33]	2017	Indonesia	Pediatric outpatient clinic/ pediatric emergency department/ inpatient pediatrics ward	6 m-5y	OMRON Gentle Temp 510	Rectal
Chatproedprai et al [3]	2016	Thailand	Pediatric outpatient clinic	0-2y	Microlife IR1DE1–1	Rectal
Acikgoz et al [30]	2016	Turkey	pediatric emergency department	3 m-3y	Genius™ 2	Rectal
Allegaert et al [5]	2014	Belgium	Pediatric wards	2 m-17y	Genius™ 2	Rectal
Teller et al [34]	2014	Swizerland	Pediatric practice	1 m-2y	Braun Thermoscan 6022™	Rectal
Hamilton et al [15]	2014	America	The emergency department and the overflow patient treatment areas	0-18y	Braun Welch Allyn Pro 4000 Thermoscan	Rectal
Batra et al [29]	2013	India	The pediatric emergency room	2-12y	Equinox infrared ear thermometer (EQ. ET 99)	Rectal
Duru et al. [31]	2012	Nigeria	The neonatal wards	6.63 ± 6.98d	Braun IRT 4520 Thermoscan	Rectal
Edelu et al [35]	2011	Nigeria	Pediatric outpatient clinic/ pediatric emergency department	0-5y	OMRON instant ear thermometer model MC-509 N	Rectal
Paes et al [8]	2010	Netherlands	The pediatric ward	0-18y	The FirstTemp Genius tympanic thermometer 3000A	Rectal
Oyakhirome et al [32]	2010	Gabon	The outpatient department	0-10y	Braun 6022 Thermoscan	Rectal

**Table 2:** Statistical characteristics of including studies

Studies	Sample	Cut-off	TP	FP	FN	TN	Se	Sp	PPV	NPV
Mogensen et al [13]	995	37.8	372	76	20	527	95	87	83	96
		38.0	350	36	43	566	89	94	91	93
Paramita et al [33]	90	37.4	65	14	3	8	96	36	82	73
		37.5	64	11	4	11	94	50	85	73
		37.6	63	11	5	11	93	50	85	69
		37.7	62	9	6	13	91	60	87	68
		37.8	60	6	8	16	88	73	91	66
Chatproedprai et al [3]	312	37.0	181	17	22	92	89	84	91	81
		37.6	126	1	77	108	62	99	99	58
Acikgoz et al [30]	354	37.25	163	22	33	136	83	86	88	80
Allegaert et al [5]	294	38.0	5	0	17	272	22	100	100	94
Teller et al [34]	254	38.0	72	4	28	150	72	97	95	84
		37.6	93	25	7	129	93	84	79	95
Hamilton et al [15]	205	38.0	87	8	6	104	94	93	92	95
Batra et al [29]	100	38.0	49	1	1	49	98	98	98	98
Duru et al [31]	300	37.5	34	3	11	252	76	99	93	97
Edelu et al [35]	710	37.8	316	10	39	345	89	97	97	90
	90	37.6	33	4	12	41	73	91	89	77
Paes et al [8]	100	38.0	20	2	5	73	80	97	91	94
Oyakhirome et al [32]	835	38.0	337	19	112	357	75	95	94	76

### Accuracy of tympanic thermometry in children under different cut-offs

The 12 studies involved 4639 children. The cut-off points were various. Among the included articles, 7 [5, 8, 18, 33–36] studies set the optimal cut-off and the other 5 [3, 13, 14, 20, 21] studies analyzed the diagnostic test accuracy of tympanic thermometry under different cut-offs. The range of the cut-off point was from 37.0 °C to 38.0 °C. Studies had data under same cut-off were synthesized.

### Accuracy under the cut-off of 37.0 °C

There was only one study [3] reported diagnostic test accuracy under the cut-off 37.0 °C. In this study, for ear temperature (37.0 °C), sensitivity, specificity, PPV, and NPV were 0.89, 0.84, 0.91, and 0.81 respectively.

### Accuracy under the cut-off of 37.25 °C

Only one study [34] gave the optimal cut-off 37.25 °C and sensitivity, specificity, PPV, and NPV were 0.83, 0.86, 0.88, and 0.80 respectively.

### Accuracy under the cut-off of 37.4 °C

There was only one study [20] reported diagnostic test accuracy under the cut-off 37.4 °C. In this study, for ear temperature (37.4 °C), sensitivity, specificity, PPV, and NPV were 0.96, 0.36, 0.82, and 0.73 respectively.

### Accuracy under the cut-off of 37.5 °C

The cut-off 37.5 °C was used in 2 studies [20, 35] and a total of 390 pediatric patients were involved. The pooled sensitivity was 0.87 (95% CI 0.79–0.92) and heterogeneity between the articles was high: 87.5% (X^2^ = 8.02, *P* < 0.05). The pooled specificity was 0.95 (95% CI 0.92–0.97) and heterogeneity between the articles was high: 97.9% (X^2^ = 47.74, *P* < 0.05).

### Accuracy under the cut-off of 37.6 °C

**Fig. 4:**
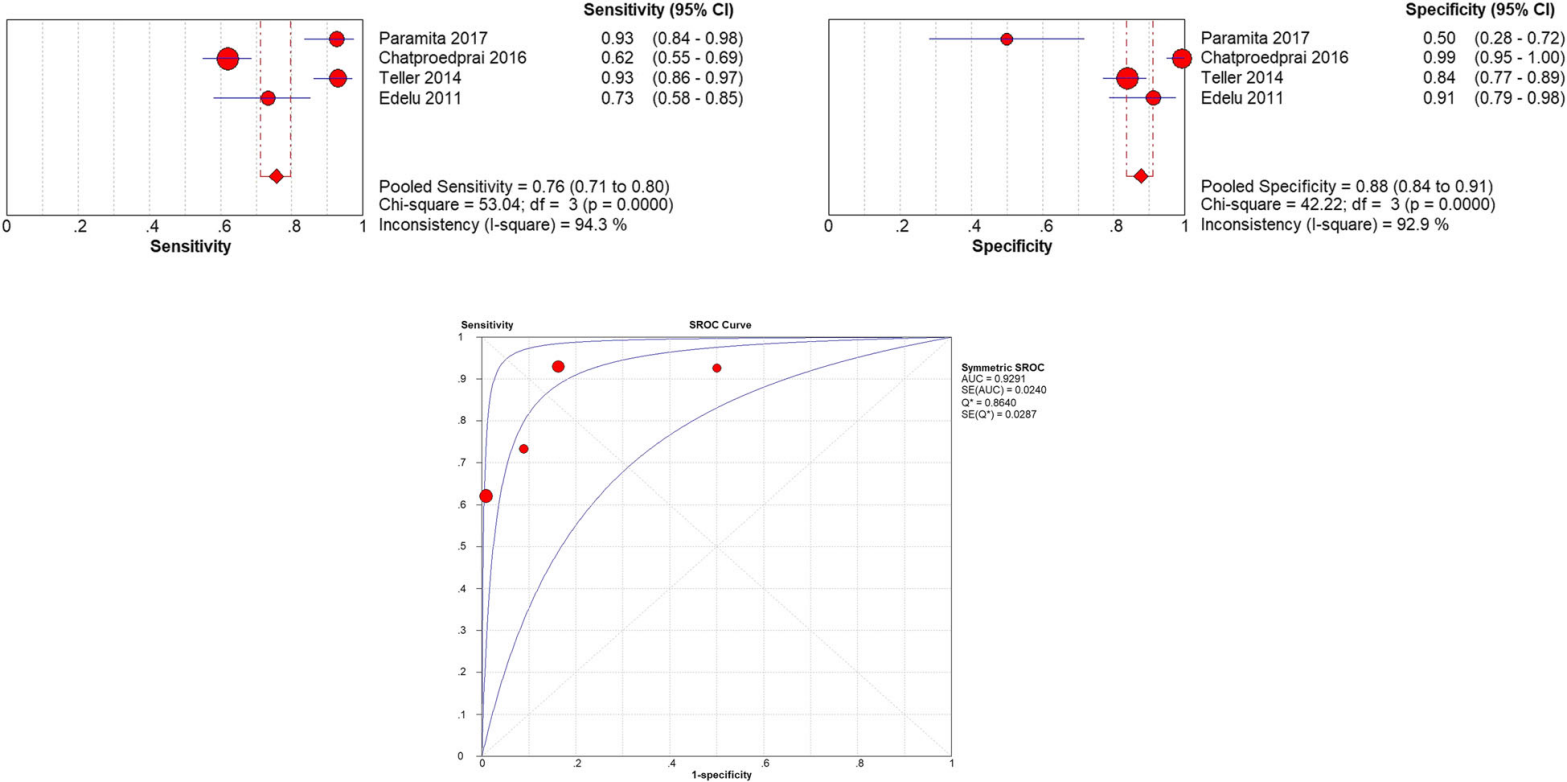
**a** The pooled sensitivity of tympanic Thermometry in Children under cut-off 37.6 °C. **b** The pooled specificity of tympanic Thermometry in Children under cut-off 37.6 °C. **c** The sROC Curve of tympanic Thermometry in Children under cut-off 37.6 °C

The cut-off 37.6 °C was used in 4 studies [3, 13, 20, 21] and a total of 746 pediatric patients were involved. Spearman’s correlation coefficient of sensitivity and specificity was 0.089 (*P* = .638) and the ROC plane showed no curvilinear trend, suggesting that there was no heterogeneity from threshold effect. The pooled sensitivity was 0.76 (95% CI 0.71–0.80) and heterogeneity between the articles was high: 94.3% (X^2^ = 53.04, *P* < 0.05). The pooled specificity was 0.88 (95% CI 0.84–0.91) and heterogeneity between the articles was high: 92.9% (X^2^ = 42.22, P < 0.05) (Fig. 4). The sROC AUC was 0.93 (SE = 0.02) while Q* value was 0.86 (SE = 0.03).

### Accuracy under the cut-off of 37.7 °C

There was only one study [20] reported diagnostic test accuracy under the cut-off 37.7 °C. In this study, for ear temperature (37.7 °C), sensitivity, specificity, PPV, and NPV were 0.91, 0.60, 0.87, and 0.68 respectively.

### Accuracy under the cut-off of 37.8 °C

**Fig. 5:**
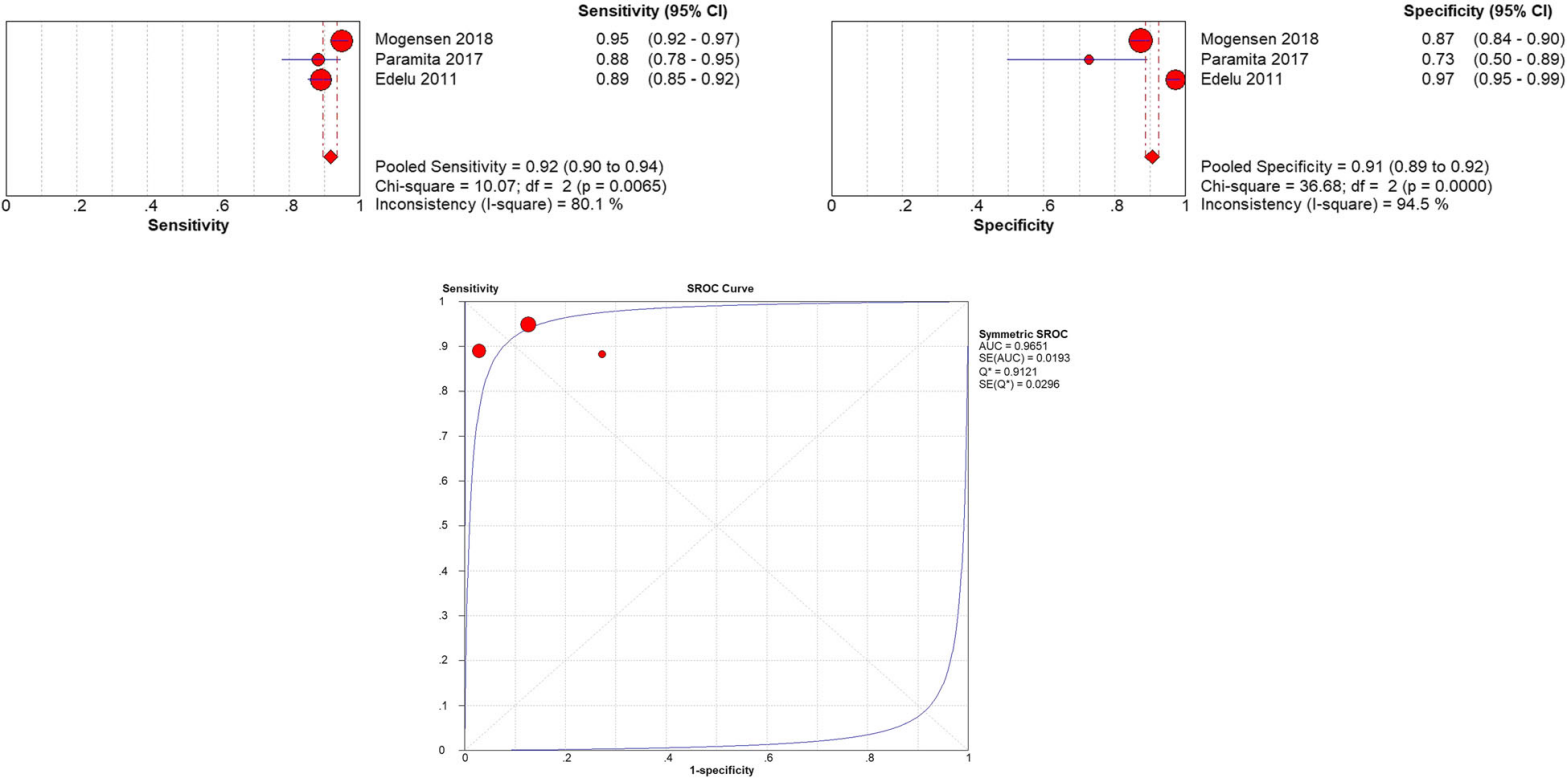
**a** The pooled sensitivity of tympanic Thermometry in Children under cut-off 37.8 °C **b** The pooled specificity of tympanic Thermometry in Children under cut-off 37.8 °C **c** The sROC Curve of tympanic Thermometry in Children under cut-off 37.8 °C

The cut-off 37.8 °C was used in 3 studies [14, 20, 21] and a total of 1795 pediatric patients were involved. The threshold analysis (*r* = − 0.050, *P* = .667) and the ROC plane (Figure) suggested that there was no heterogeneity from threshold effect. The pooled sensitivity was 0.92 (95% CI 0.90–0.94) and heterogeneity between the articles was high: 80.1% (X^2^ = 10.07, *P* < 0.05). The pooled specificity was 0.91 (95% CI 0.89–0.92) and heterogeneity between the articles was high: 94.5% (X^2^ = 36.68, *P* < 0.05) (Fig. 5). The sROC AUC was 0.97 (SE = 0.02) while Q* value was 0.91 (SE = 0.03).

### Accuracy under the cut-off of 38.0 °C

**Fig. 6:**
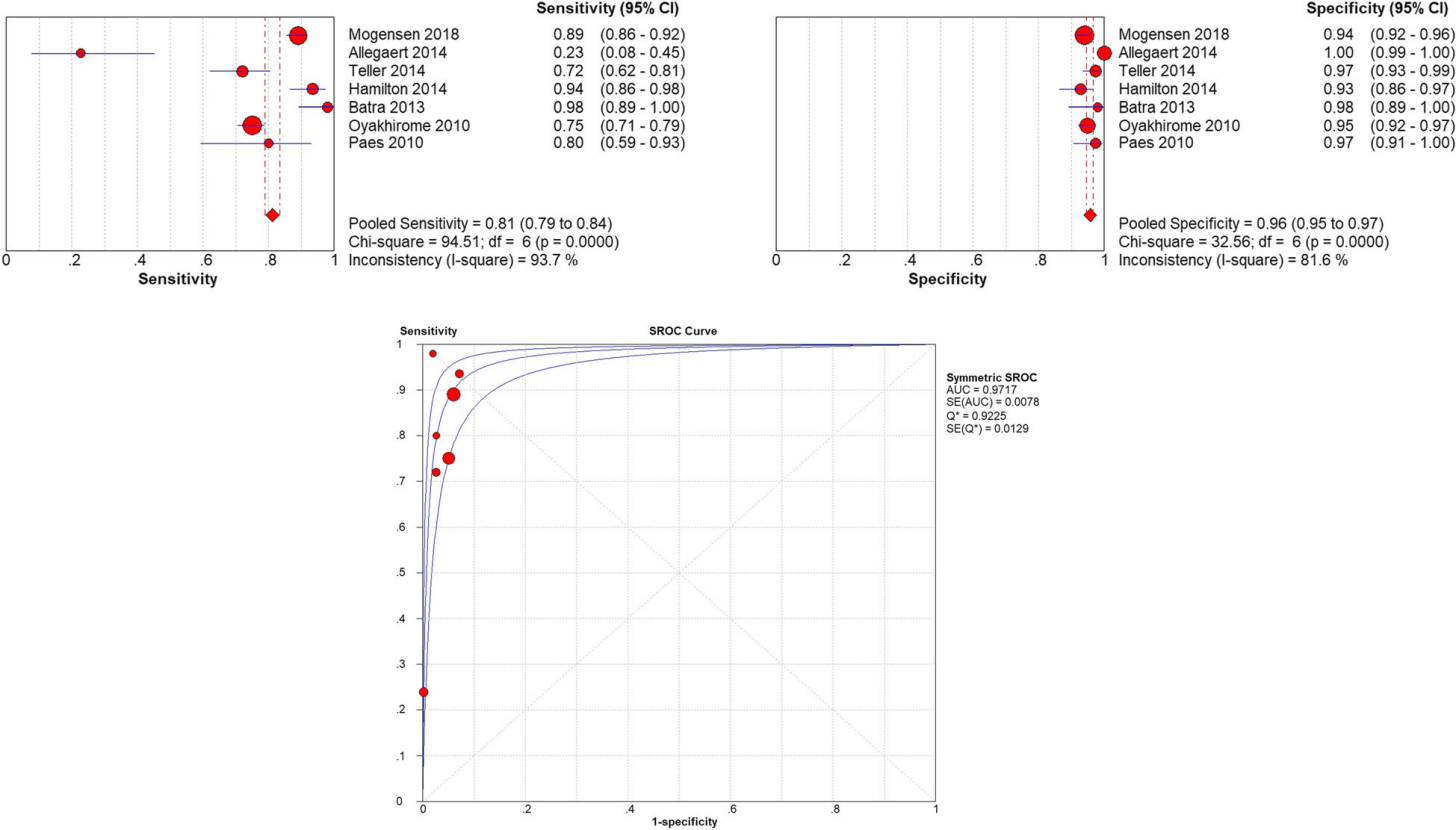
**a** The pooled sensitivity of tympanic Thermometry in Children under cut-off 38.0 °C. **b** The pooled specificity of tympanic Thermometry in Children under cut-off 38.0 °C. **c** The sROC Curve of tympanic Thermometry in Children under cut-off 38.0

The cut-off 38.0 °C was used in 7 studies [5, 8, 13, 14, 18, 33, 36] and a total of 2783 pediatric patients were involved. The threshold analysis (r = 0.429, *P* = 0.337) and the ROC plane suggested that there was no heterogeneity from threshold effect. The pooled sensitivity was 0.81 (95% CI 0.79–0.84) and heterogeneity between the articles was high: 93.7% (X^2^ = 94.51, *P* < 0.05). The pooled specificity was 0.96 (95% CI 0.95–0.97) and heterogeneity between the articles was high: 81.6% (X^2^ = 32.56, *P* < 0.05) (Fig. 6). The sROC AUC was 0.97 (SE = 0.01) while Q* value was 0.92 (SE = 0.01).

The diagnostic test accuracy of the tympanic thermometry under different Cut-offs in the detection of pediatric fever is summarized in Table 3. The cut-off 37.8 is with the highest sROC AUC and Youden Index and is deemed to be the optimal cutoff.

**Table 3:** Accuracy of tympanic thermometry under different cutoffs in children

Cut-off(°C)	N	Sen	Sp	Youden Index	SROC-AUC
37.0	312	89	84	0.73	N
37.25	354	83	86	0.69	N
37.4	90	96	36	0.32	N
37.5	390	87	95	0.82	N
37.6	746	76	88	0.64	0.93
37.7	90	91	60	0.51	N
37.8	1795	92	91	0.83	0.97
38.0	2578	80	96	0.76	0.97

## Discussion

We conducted this study to assess the discriminant validity of the new generation IRTT for detecting pediatric fever determined by rectal thermometry and to find the optimal cutoff. Twelve studies, including 4639 children, were included. The results indicated that IRTT was a good alternative for rectal thermometry in pediatric patients, and the optimal cut-off of ear temperature for screening fever in children was 37.8 °C. Under this cut-off, pooled sensitivity was 0.92 (95% CI 0.90–0.94), pooled specificity was 0.91 (95% CI 0.89–0.92), sROC AUC was 0.97 (SE = 0.02) and Q* value was 0.91 (SE = 0.03).

One major strength of this study was that it estimated the test accuracy of new generation IRTT. Although the IRTT may provide a good alternative for traditional measurements, it has been debated for the low reproducibility. However, since the ear thermometer came out, it has been constantly updated and upgraded. Some techniques have been used to improve the test accuracy, such as the Braun Welch Allyn Pro 4000 Thermoscan, where a heating element in the sensor heats the probe tip to just below normal body temperature to avoid cooling the ear canal [19]. And the improvements of geometry and algorithms have been developed to ensure that the displayed result reflects the tympanic temperature accurately [11]. Hence, the newer versions of tympanic thermometers might meet the clinicians’ requested improvements of repeatability in noninvasive temperature assessments. By new generation, we mean the IRTT that were still in production and on sale according to the official websites of the manufacturers as we started our study. We included the tympanic thermometers under use and excluded the outdated ones so that the results could provide a reference for current clinical practice.

Another strength of this study was that it estimated the test accuracy of new generation IRTT under different cutoffs. The synthesis of data under different cutoffs may underestimated the test accuracy of IRTT, because the diagnostic accuracy of IRTT varied under different cutoffs [3, 13, 20, 21]. The cutoffs of IRTT ranged from 37.0 °C to 38 °C among these 12 included studies. After the synthesis of three studies, including 1795 children, we found the optimal cut-off of tympanic thermometry is 37.8 °C. And under this cutoff, the pooled sensitivity was 0.92 (95% CI 0.90–0.94), pooled specificity was 0.91 (95% CI 0.89–0.92), sROC AUC was 0.97 (SE = 0.02) and Q* value was 0.91 (SE = 0.03).

The diagnostic accuracy in this study under the optimal cutoff was far higher than a former systematic review [27], in which pooled sensitivity was 0.70 (95% CI 0.68–0.72), pooled specificity was 0.86 (95% CI 0.85–0.88), sROC AUC was 0.94, and Q* value was 0.87. Excluding articles applying obsolete tympanic thermometers and analyzing diagnostic test accuracy under different cut-offs may be the major reasons for this gap.

The 12 included studies are with high homogeneity, because they have the same study type, study population, reference standard and et al. And data were synthesized by using the random-effects model. What should be underlined is that the heterogeneity between the articles is very high, from 81.6 to 94.5%. The study population of included studies are all children, who age from 0 to 18-year-old. But the age groups are various, for example, Duru et al. [35] admitted neonates whose mean age is 6.63 ± 6.98 days, while Allegaert et al. [5] enrolled children with a median age of 3.2 years (range 0.02 years to 17 years). The variation of age groups may be the major contribution to the high heterogeneity and further studies focusing on different age groups are needed.

Although the results of our study can provide an important reference for subsequent researches and clinical applications, there are two limitations in our present study. We performed different sub-group meta-analyses based on the different cut-offs used. Unfortunately, in many of these analyses a limited number of studies are included. We concluded that 37.8 °C was the optimal cut-off just based on three studies, which seemed unconvincing. But considering that 1795 subjects were included for analysis under the cut-off 37.8 °C, the conclusion was much more convincing.

According to the findings, ear canal temperature can be confidently implemented as a screening measure in the pediatric fever detection. This application of IRTT would effectively decrease the number of children who require the rectal temperature method for fever detection [7]. However, there are some situations, such as uncertain diagnosis [7], during exercise [37, 38], change of environmental temperatures [39], that tympanic temperature should not be used as a surrogate for rectal temperature.

## Conclusion

Tympanic thermometry has a high diagnostic accuracy and is a good alternative for temperature screening in pediatric patients. The optimal cut-off of ear temperature for screening fever in children is 37.8 °C. Tympanic thermometry may not be an alternative for rectal temperature after intense exercise or exertion heat stroke.

## Data Availability

Not applicable.

## Abbreviations

IRTT: Infrared tympanic thermometer
FP: The false Positive
FN: The false Negative
NPV: Negative predictive value
PPV: Positive predictive value
The PRISMA-DTA Statement: the Preferred Reporting Items for Systematic Reviews and Meta-Analyses of Diagnostic Test Accuracy Studies
QUADAS-2: The Quality Assessment of Diagnostic Accuracy Studies-2
TP: The True Positive
TN: The True Negative
